# Laser weeding of common weed species

**DOI:** 10.3389/fpls.2024.1375164

**Published:** 2024-05-24

**Authors:** Christian Andreasen, Eleni Vlassi, Najmeh Salehan

**Affiliations:** Department of Plant and Environmental Sciences, Faculty of Science, University of Copenhagen, Taastrup, Denmark

**Keywords:** integrated weed management, laser weeding, non-chemical weed control, sitespecific weed management, thermal weed control

## Abstract

The massive use of herbicides since the 1950s has resulted in increasing problems with herbicideresistant weeds and pollution of the environment, including food, feed, and water. These side effects have resulted in political pressures to reduce herbicide application. The European Commission aims to reduce the use and risk of chemicals and more hazardous pesticides in the EU. Therefore, new weed control methods are in demand. Laser weeding might be an alternative to replace or supplement herbicides and other weed control methods in an Integrated Weed Management (IPM) strategy. This work aimed to investigate how increasing laser energy affected common weeds when the apical meristem was exposed to irradiation at the early stages of development. A 50 W thulium-doped fibre laser with a diameter of 2 mm and a wavelength of 2 µm was used. The highest efficacy of laser irradiation was achieved when the grass weed (*Alopecurus myosuroides*) had one leaf and the dicot species were at the cotyledon stage. There was a large difference between the species’ susceptibility to irradiation probably caused by differences in morphology and growth habit. At the 4-leaf stage, most of the species regrew after irradiation. Laser weeding may be a solution to replace or supplement other weed control methods in some crops, but in general the weeds must be irradiated when they are at the cotyledon to 2-leaf stage to avoid regrowth.

## 1 Introduction

Weeds are one of the main constraints for crop production, and herbicides are the dominant tool to control weeds in modern agriculture. The massive use of herbicides has resulted in the evolution of herbicide-resistant weeds (Beckie, 2006; Heap, 2024). Herbicide use has led to unintentional pollution of feed, food, and the environment (Silva et al., 2019; Rani et al., 2021), and, therefore, strict regulations for pesticide application have been implemented by the EU and in many other countries to reduce the adverse side effects (Kudsk and Mathiassen, 2020). The European Commission aims to reduce the use and risk of chemicals and more hazardous pesticides in the EU (Silva et al., 2022). Site-specific herbicide applications can reduce the adverse side effects, but they could be eliminated if herbicides could be replaced with other methods.

Mechanical weed control is practised on organic farms and in combination with herbicide application on some conventional farms. However, mechanical weeding may also harm living organisms like beneficial insects on the soil surface (e.g., predatory beetles and spiders) and earthworms in the soil (Tamburini et al., 2016; Sharma et al., 2017). It may also create soil erosion, dry out soils with limited moisture content, promote unnecessary mineralization of soil organic matter, and cause leaching of plant nutrients. Furthermore, it stimulates new cohorts of weeds seeds to germinate (Cloutier and Leblanc, 2001). The negative impact on the environment can likely be reduced by practicing site-specific weed harrowing (Berge et al., 2024).

Since the 2000’s, laser has been considered a potential method to control weeds (Heisel et al., 2001, 2002), but laser is energy demanding, and, therefore, it is essential solely to spend the energy on the weed plants. Electricity is a precondition for lasers. It can be provided by an engine or batteries, for example, supplied from renewable energy sources. The fast development in computer vision and artificial intelligence has now opened new promising perspectives for laser weeding (Rakhmatulin et al., 2021; Andreasen et al., 2022). Identification of plant species and recognition of the location can be done rapidly and precisely (Rakhmatulin et al., 2021) and a laser can be guided by mirrors to target weed in the apical meristem and kill it with heat (Rakhmatulin and Andreasen, 2020; Coleman et al., 2021). With a laser beam diameter of 2 mm and 300 weeds m^-2^, the total area exposed to the treatment will be 300 × 22/7 × 1^2^ mm^2 =^ 314.3 mm^2^, corresponding to less than 0.1% of the area. This is the most site-specific weed management which can be achieved.

A laser beam might harm non-target organisms on plants, and the ground (Mullen et al., 2016; Andreasen et al., 2023), but the risk is very low as the target area is tiny. The mortality of earthworms seems not to increase when the soil is exposed to the laser beam (Andreasen et al., 2023).

It is necessary to study the relationship between laser dose and the effect on the weeds to avoid overuse of energy. This paper reports experiments with a 50 W thulium-doped fibre laser with a diameter of 2 mm and a wavelength of 2 µm. The laser type was intended to be mounted on an autonomous field vehicle to control weed seedlings (Emmi et al., 2024) and was developed in the EU project WeLASER (https://welaser-project.eu/). The fibre laser was chosen because it is assumed to be more efficient than a CO_2_ laser for controlling weeds. While a fibre laser with a 2 µm wavelength penetrates the plant cells and heats the water inside the cells, the energy from the CO_2_ laser is mainly absorbed on the surface of the plant cells (Wieliczka et al., 1989; Andreasen et al., 2022). This project aims to investigate how laser affected the growth and development of a grass weed, a crop, and some common dicotyledonous weed species when they were exposed to increasing doses of energy at the apical meristem in the early stages of development.

## 2 Materials and methods

### 2.1 Laser equipment

**Figure 1:**
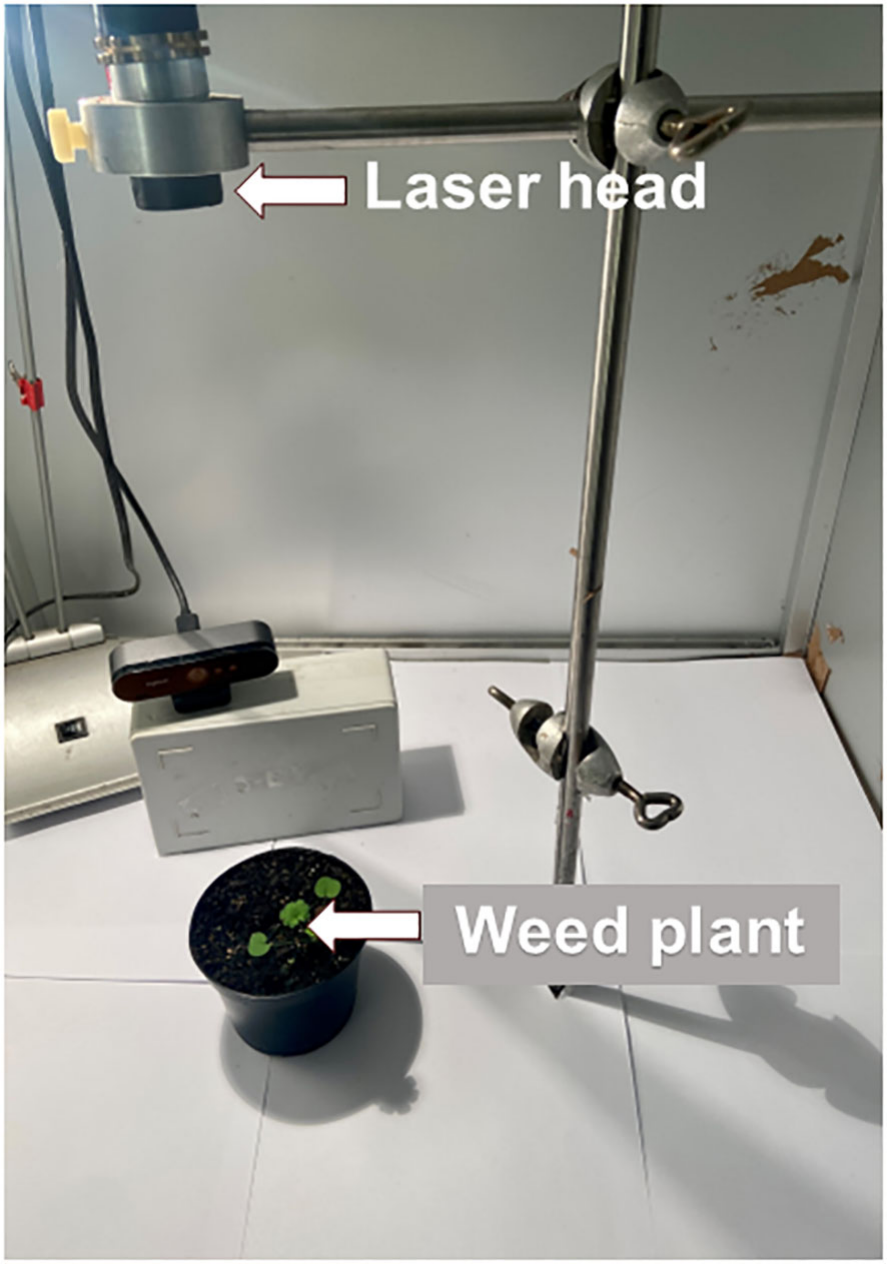
Cabinet with mounted laser used for dose-response experiments.


A thulium-doped 50 W fibre laser with a wavelength of 2 µm and a collimated beam (Ø: 2 mm) were used. Futonics Laser GmbH, Katlenburg-Lindau, Germany, manufactured the laser equipment. The laser head was placed within a steel box (68 cm × 68 cm × 68 cm) with a door with a metal interlock (Andreasen et al., 2023) (**Figure 1**). The door locked automatically when the laser was activated to avoid exposing users to reflections. The laser dose was determined and activated from a computer. Plants were placed approximately 30−35 cm from the laser head and exposed to increasing dosages of laser energy up to 12.7 J mm^-2^. The dose was determined by the time (s) the target was exposed to the irradiation, and the energy consumption was calculated using Equation 1.

(1) $$\text{Dose }\left(\text{J }{\text{mm}}^{-2}\right)=50\text{ W}\times \text{s}/\left(22/7\times 1^{2}{\text{mm}}^{2}\right)$$

### 2.2 Plant species

Species included *Alopecurus myosuroides* Huds., a winter annual grass causing increasing problems in Northern Europe, particularly in autumn sown crops like barley, rye, and wheat (Menchari et al., 2007). Sugar beet (*Beta vulgaris* L. cv. Bangor) was selected as a suitable row crop for laser weeding (Andreasen et al., 2022), but it also occurs as a volunteer in following crops. Additionally, the dicot species *Erodium cicutarium* (L.) Hér, *Geranium molle* (L.), *Lamium purpureum* L., *Myosotis arvensis* (L.) Hill, *Plantago major* L., *Rumex crispus* L., *Stellaria media* (L.) Kuntze, *Sonchus oleraceus* L., *Veronica persica* Poir, and *Viola arvensis* Murray were chosen due to their prevalence in both summer and winter annual crops in Europe (Rydberg and Milberg, 2000; Andreasen and Stryhn, 2012; Kraehmer et al., 2020).

All weed seeds used in the experiments were produced from plants collected in fields belonging to the University of Copenhagen, Højbakkegaard, Taastrup (55° 38’ N, 12° 17’E), Denmark, except beet seeds.

For each species, 5−15 seeds were sown in 21 plastic pots (height: 7 cm; Ø: 10 cm) containing a sphagnum soil [Pindstrup Ready Mix 2 (https://www.pindstrup.dk/professionel/product-details/pindstrup-f%C3%A6rdigblanding-2)] in a greenhouse. The first time the pots were irrigated after sowing, they were watered with the insecticide Gnatrol SC ^®^ (Nordic Alkali, Anemonevænget 2, DK 4330 Hvalsø, Denmark) containing the biocontrol agent *Bacillus thuringiensis* subsp. *israelensis* AM65–52 (1.8 x 1011 CFU/l (11,6% (w/w)) corresponding to 123 g L^-1^ to prevent fungus gnats (*Bradysia coprophila* and *Bradysia impatiens*). After sowing, the pots were randomly placed in plastic trays (58 cm × 30 cm) with holes in the bottom to facilitate irrigation. The irrigation was carried out daily by applying water through the bottom of the tray, ensuring that lack of water was not a growth factor. After emergence, plants were thinned to one plant per pot. When the plants had obtained the desired growth stage, they were moved to the laser cabinet and irradiated.

*Alopecurus myosuroide*s plants were irradiated when they had one, two, or three leaves, respectively. In a pre-experiment the plants were irradiated from an angle of 90° (data not shown), but because it did not affect the grass much, the plants were irradiated close to the soil from an angle of 45°.

The dicots were treated when they had developed two cotyledons, two permanent leaves, or four permanent leaves, respectively. The apical meristem was irradiated from above (90°).

Three plants of each weed species were exposed to one of each of the following doses: 0 s, 0.025 s, 0.05 s, 0.1 s, 0.2 s, 0.4 s, and 0.8 s corresponding to 0 J mm^-2^, 0.4 J mm^-2^, 0.8 J mm^-2^, 1.6 J mm^-2^, 3.2 J mm^-2^, 6.4 J mm^-2^, and 12.7 J mm^-2^. Hence, each dose-response experiment consisted of 3 pots × 7 doses = 21 pots. The plants were moved back to the greenhouse after the treatment. After 21 days, the plants were cut just above the ground, and the fresh weight was measured. The experiments were conducted between June and December 2023 (**Table 1**). In total, 1512 plants were included (3 replicates × 7 doses × 3 developmental stages × 2 experiments × 12 species).

**Table 1:** The experiment periods, which took place from 5 July to 15 December 2023.

		Experimental period (date/month)		
Monocot	Exp.	One leaf	Two leaves	Three leaves
*Alopecurus myosuroides*	1	5/7−−26/7	17/7−7/8	14/7− 4/8
*Alopecurus myosuroides*	2	14/4−7/5	23/6−14/7	19/6−13/7
Dicots		Cotyledon stage	Two permanent leaves	Four permanent leaves
*Beta vulgaris*	*1*	18/7−8/8	26/7−16/8	11/8−1/9
*Beta vulgaris*	*2*	22/8−12/9	31/8−21/9	7/9−28/9
*Erodium dissectum*	*1*	24/8−14/9	18/10−8/11	28/8−18/9
*Erodium dissectum*	*2*	8/9−29/9	18/10−8/11	2/10−23/10
*Geranium molle*	*1*	16/8−6/9	22/8−12/9	30/8−20/9
*Geranium molle*	*2*	22/9−13/10	30/10−20/11	10/10−31/10
*Lamium purpureum*	*1*	3/8−24/8	11/8−1/9	22/8−12/9
*Lamium purpureum*	*2*	20/9−11/10	24/11−15/12	17/10−7/11
*Myosotis arvensis*	*1*	24/7−14/8	28/7−18/8	11/10−1/11
*Myosotis arvensis*	*2*	4/8−25/8	23/8−13/9	27/10−17/11
*Plantago major*	*1*	1/8−22/8	12/7−2/8	20/7−10/8
*Plantago major*	*2*	15/8−5/9	8/8−29/8	6/10−27/10
*Rumex crispus*	*1*	16/8−6/9	23/8−13/9	7/9−28/9
*Rumex crispus*	*2*	24/8−14/9	1/9−22/9	30/8−20/9
*Sonchus oleraceus*	*1*	3/7−24/7	10/7−31/7	27/9−18/10
*Sonchus oleraceus*	*2*	2/8−23/8	3/10−24/10	29/8−19/9
*Stellaria media*	*1*	24/7−14/8	6/7−27/7	28/8−18/9
*Stellaria media*	*2*	10/8−31/8	3/8−24/8	29/8−19/9
*Veronica persica*	*1*	27/6−17/7	11/7−1/8	18/7−8/8
*Veronica persica*	*2*	19/9−10/10	28/8−18/9	4/9−25/9
*Viola arvensis*	*1*	11/8−1/9	29/8−19/9	10/10−31/10
*Viola arvensis*	*2*	6/9−27/9	12/10−2/11	24/10−14/11

*Alopecurus myosuroides* plants were exposed to laser at the one, two, and three 1eaf stages, respectively. Dicotyledonous plants were irradiated at the cotyledon, two permanent leaf, and four permanent leaf stage, respectively.

### 2.3 Statistical analyses

All data sets were individually analyzed, as the experiments were independent and done during a long period with varying temperature and light conditions (**Table 1**). The response, *y*, is described by a log-logistic dose-response curve. The distribution of residuals and the fit themselves were used to assess the quality of the regressions using the statistical software R version 4.2.0 (R Core Team, 2022) with the add-on drc package (version 4.2.3). A three-parameter model was used to describe the data (Equation 2):


(2) $$y=\frac{d}{1+\text{exp}[b\left(\text{log}(x\right)-\log\left(e\right))]\;}$$

*y* is the biomass three weeks after the treatment, *d* is a parameter close to the untreated control (upper limit). The parameter *b* is proportional to the slope of the curve at the dose *e*, which is the effective dose that reduces the biomass by 50% (ED_50_). The effective doses, ED_10,_ ED_50,_ and ED_90_, resulting in a 10, 50, or 90 percent biomass reduction, respectively, were estimated.

## 3 Results

**Figure 2:**
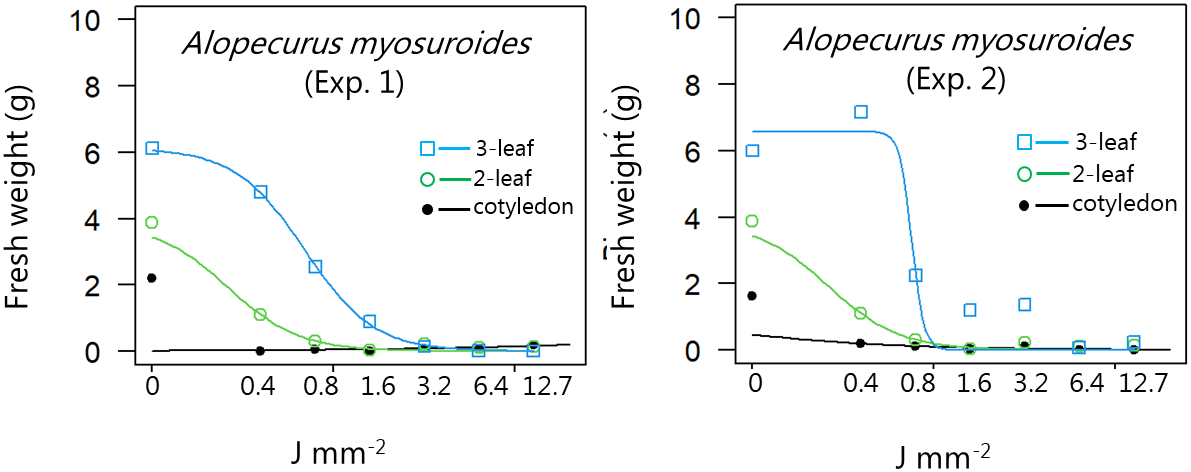
Dose-response experiment 1 and 2 with *Alopecurus myosuroides*. Fresh weight was measured 21 days after the treatment. Plant developmental stages at the time of laser treatment: • = 1-leaf, ○ = 2-leaf, □ = 3-leaf.

**Figure 3:**
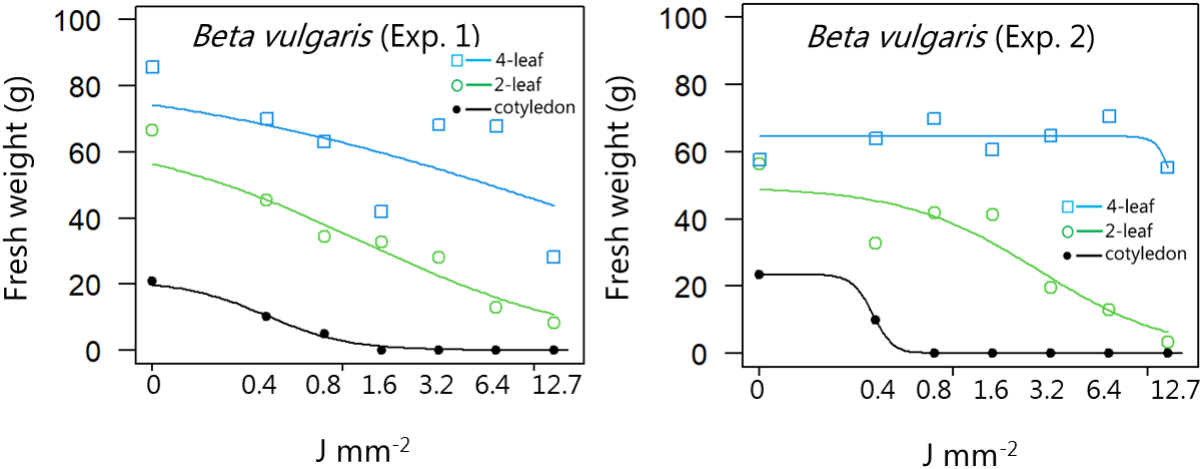
Dose-response experiment 1 and 2 with *Beta vulgaris*. Fresh weight was measured 21 days after the treatment. Plant developmental stages at the time of laser treatment: • = cotyledons, ○ = 2-leaf, □ = 4-leaf.

**Figure 4:**
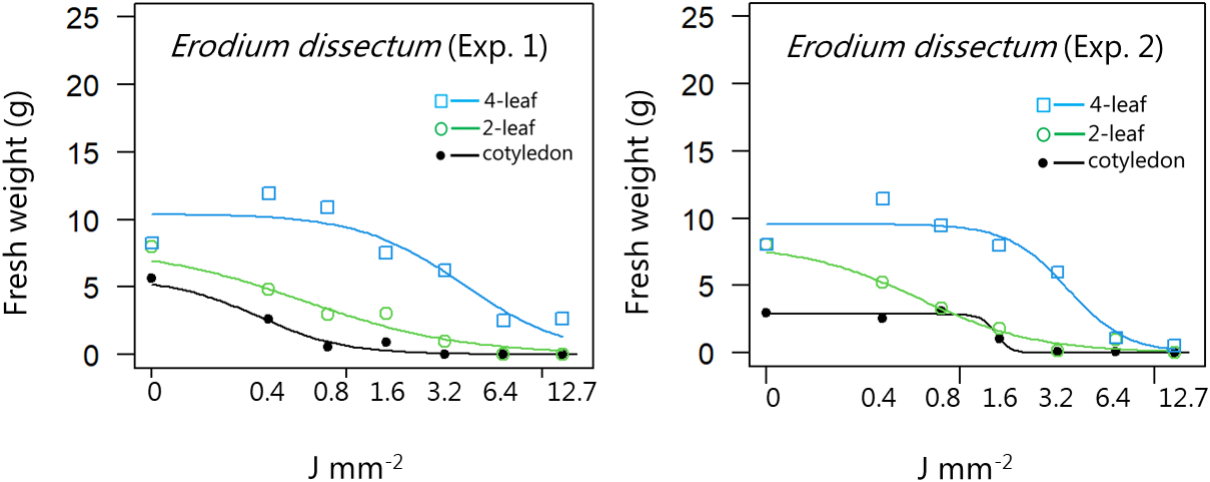
Dose-response experiment 1 and 2 with *Erodium dissectum*. Fresh weight was measured 21 days after the treatment. Plant developmental stages at the time of laser treatment: • = cotyledons, ○ = 2-leaf, □ = 4-leaf.

**Figure 5:**
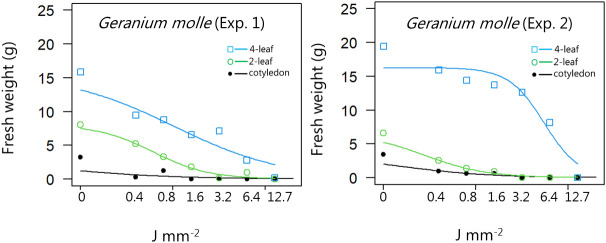
Dose-response experiment 1 and 2 with *Geranium molle*. Fresh weight was measured 21 days after the treatment. Plant developmental stages at the time of laser treatment: • = cotyledons, ○ = 2-leaf, □ = 4-leaf.

**Figure 6:**
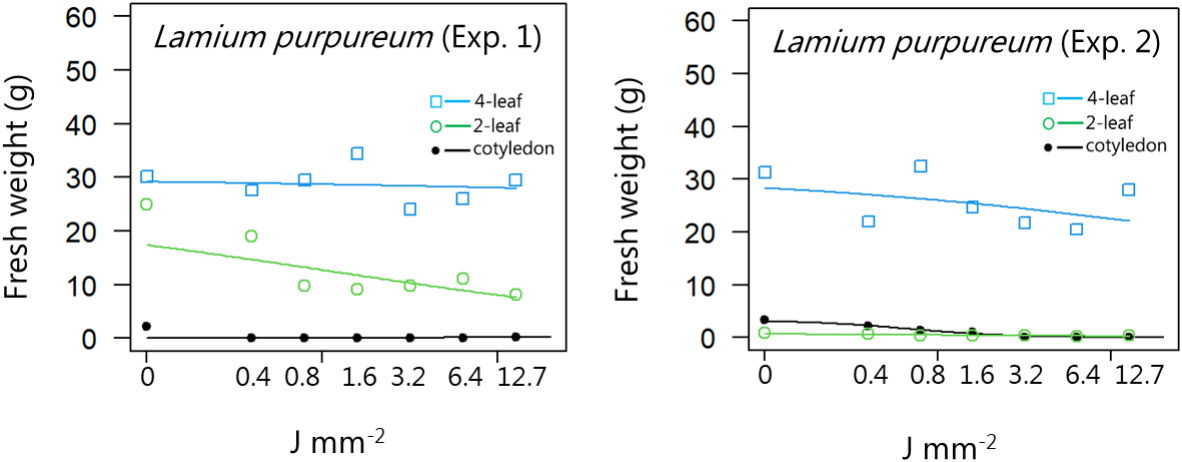
Dose-response experiment 1 and 2 with *Lamium purpureum*. Fresh weight was measured 21 days after the treatment. Plant developmental stages at the time of laser treatment: • = cotyledons, ○ = 2-leaf, □ = 4-leaf.

**Figure 7:**
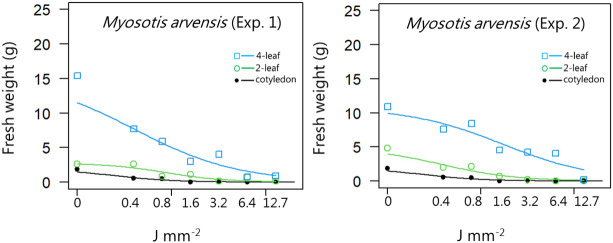
Dose-response experiment 1 and 2 with *Myosotis arvensis*. Fresh weight was measured 21 days after the treatment. Plant developmental stages at the time of laser treatment: • = cotyledons, ○ = 2-leaf; □ = 4-leaf.

**Figure 8:**
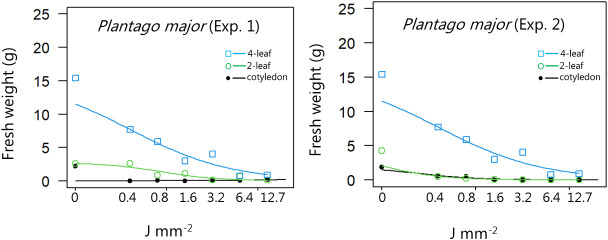
Dose-response experiment 1 and 2 with *Plantago major*. Fresh weight was measured 21 days after the treatment. Plant developmental stages at the time of laser treatment: • = cotyledons, ○ = 2-leaf, □ = 4-leaf.

**Figure 9:**
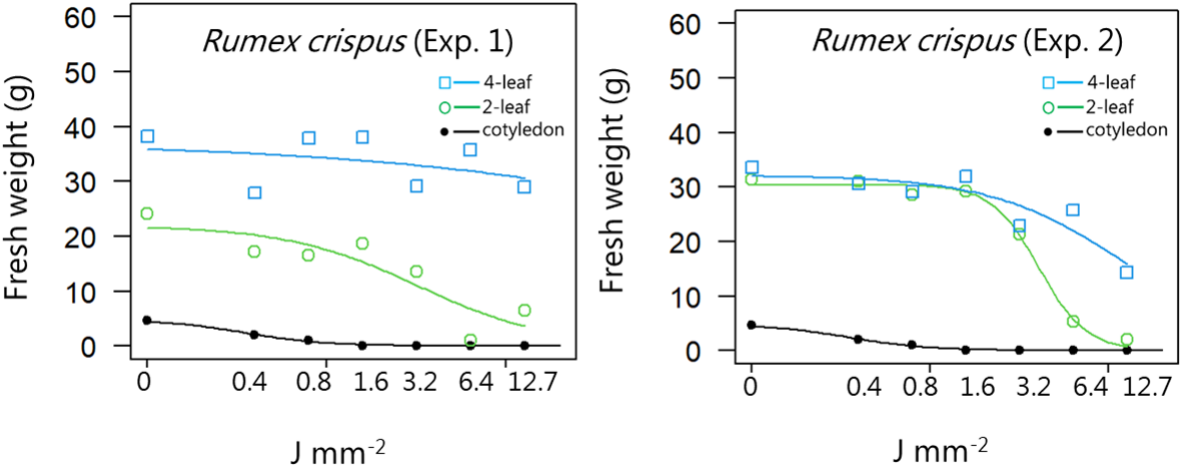
Dose-response experiment 1 and 2 with *Rumex crispus*. Fresh weight was measured 21 days after the treatment. Plant developmental stages at the time of laser treatment: • = cotyledons, ○ = 2-leaf, □ = 4-leaf.

**Figure 10:**
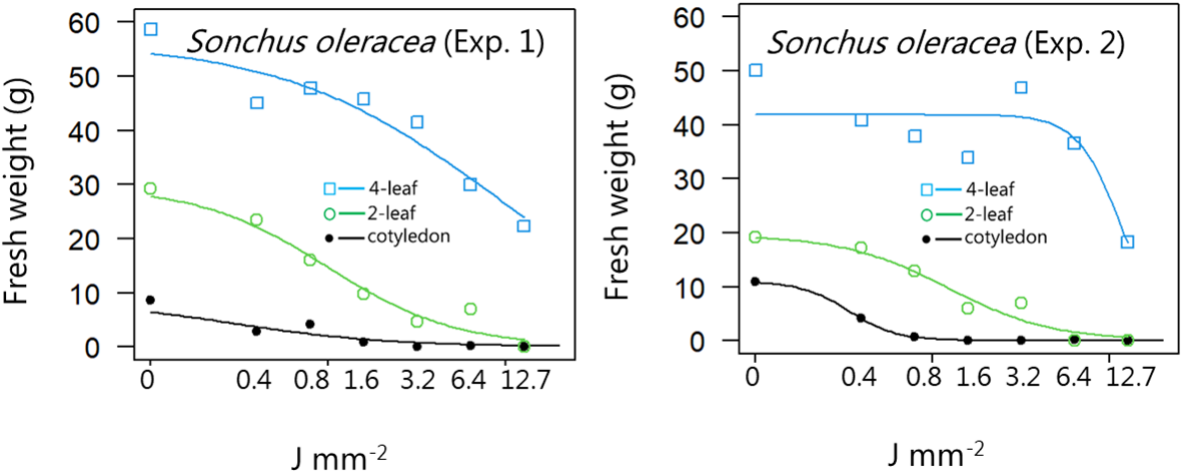
Dose-response experiment 1 and 2 with *Sonchus oleracea*. Fresh weight was measured 21 days after the treatment. Plant developmental stages at the time of laser treatment: • = cotyledons, ○ = 2-leaf, □ = 4-leaf.

**Figure 11:**
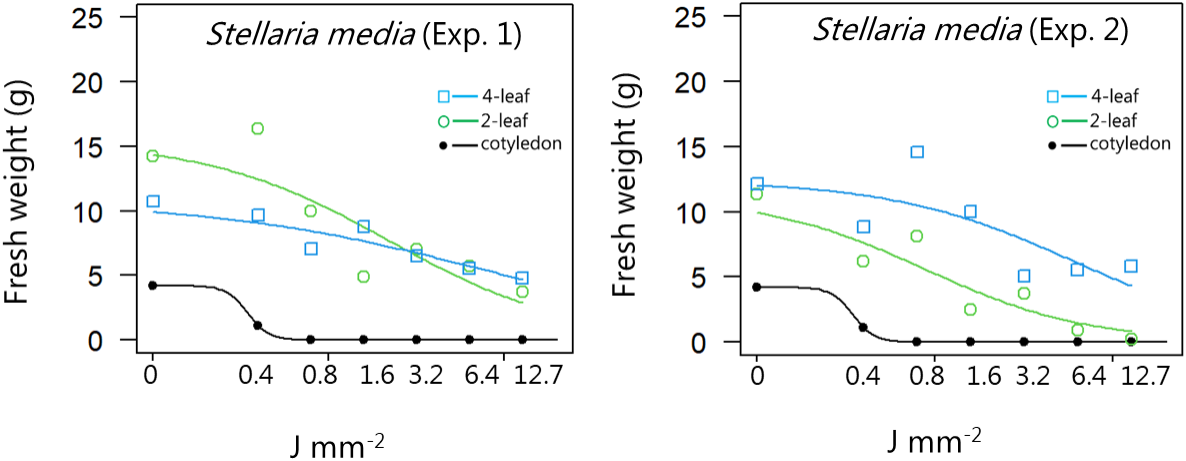
Dose-response experiment 1 and 2 with *Stellaria media*. Fresh weight was measured 21 days after the treatment. Plant developmental stages at the time of laser treatment: • = cotyledons, ○ = 2-leaf, □ = 4-leaf.

**Figure 12:**
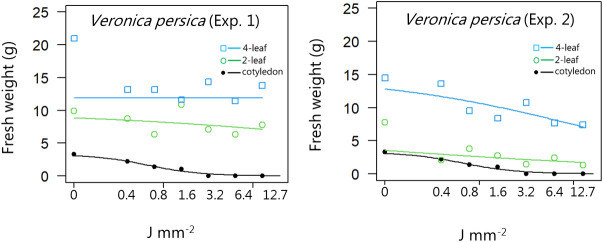
Dose-response experiment 1 and 2 with *Veronica persica*. Fresh weight was measured 21 days after the treatment. Plant developmental stages at the time of laser treatment: • = cotyledons, ○ = 2-leaf, □ = 4-leaf.

**Figure 13:**
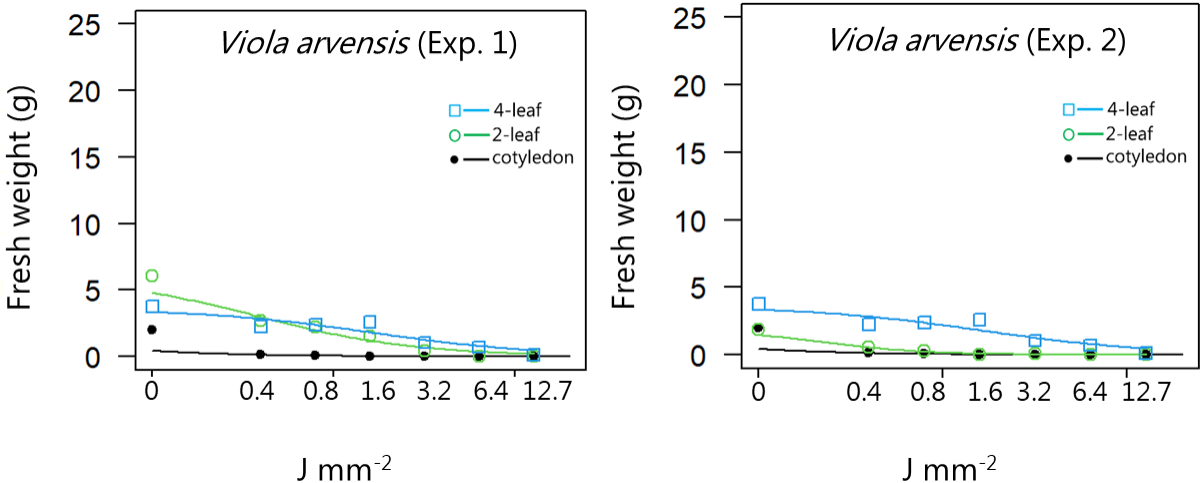
Dose-response experiment 1 and 2 with *Viola arvensis*. Fresh weight was measured 21 days after the treatment. Plant developmental stages at the time of laser treatment: • = cotyledons, ○ = 2-leaf, □ = 4-leaf.

Although the dose-response experiments were conducted under different growing conditions (**Table 1**), the results are presented in one figure for three different growth stages for each species to limit the numbers of figures (**Figures 2**–**13**). The estimated model parameters are shown with Standard Error (SE) in **Table 2** and ED_10_, ED_50_, and ED_90_ with SE in the **Table 3**. For some developmental stages, the model of the S-shape curve did not fit well because of biological variation and because even the smallest dose prohibited regrowth (e.g., *A. myosuroides* at the one-leaf stage) (**Figure 2**; **Table 2**). Still, the model gives a good overview of the trend in the results.

**Table 2:** Estimated parameters according to the dose-response model (Equation 2) for plants exposed to increasing laser doses on three growth stages in two experiments (Exp.).

	Exp.	Growth stage	*b*	*d*	*e*
*Alopecurus myosuroides*	1	One-leaf stage	-0.49 (1.78)	8.46 (137)	4 * 10^4^ (5*10^5^)
		Two-leaf stage	2.17 (0.98)	3.89 (0.18)	0.26 (0.07)
		Three-leaf stage	2.26 (0.81)	6.13 (0.67)	0.69 (0.14)
*Alopecurus myosuroides*	2	One-leaf stage	0.80 (0.66)	1.62 (0.09)	0.03 (0.08)
		Two-leaf stage	2.12 (0.98)	3.89 (0.18)	0.26 (0.07)
		Three-leaf stage	13.0 (67.0)	6.59 (0.66)	0.76 (0.20)
*Beta vulgaris*	1	Cotyledon stage	2.01 (0.82)	21.0 (2.23)	0.40 (0.09)
		Two-leaf stage	0.70 (0.21)	66.1 (7.18)	1.24 (0.58)
		Four-leaf stage	0.39 (0.25)	85.0 (11.1)	14.9 (17.5)
*Beta vulgaris*	2	Cotyledon stage	8.38 (40.7)	23.5 (1.95)	0.38 (0.07)
		Two-leaf stages	1.25 (1.19)	49.5 (12.1)	2.75 (1.90)
		Four-leaf stages	10.5 (40.5)	64.6 (2.38)	15.1 (9.91)
*Erodium dissectum*	1	Cotyledon stage	1.95 (1.30)	5.65 (0.59)	0.35 (0.10)
		Two-leaf stages	1.09 (0.33)	7.92 (0.89)	0.61 (0.22)
		Four-leaf stages	1.63 (0.69)	10.4 (1.20)	3.94 (1.35)
*Erodium dissectum*	2	Cotyledon stage	11.7 (26.9)	2.86 (0.19)	1.52 (0.17)
		Two-leaf stages	1.43 (0.35)	8.04 (0.64)	0.61 (0.12)
		Four-leaf stages	2.84 (1.47)	9.56 (0.83)	3.57 (0.70)
*Geranium molle*	1	Cotyledon stage	0.67 (0.45)	3.26 (0.33)	0.05 (0.09)
		Two-leaf stages	1.43 (0.35)	8.04 (0.64)	0.61 (0.12)
		Four-leaf stages	0.73 (0.15)	15.6 (1.23)	1.04 (0.35)
*Geranium molle*	2	Cotyledon stage	0.88 (0.38)	3.46 (0.28)	0.15 (0.11)
		Two-leaf stages	1.29 (0.44)	6.61 (0.50)	0.28 (0.09)
		Four-leaf stages	2.28 (1.25)	16.3 (1.38)	5.49 (1.29)
*Lamium purpureum*	1	Cotyledon stage	-0.49 (1.78)	8.46 (137)	4.0×10^4^ (4.9×10^5^)
		Two-leaf stages	0.34 (0.14)	25.1 (2.26)	1.09 (0.80)
		Four-leaf stages	0.16 (0.09)	30.3 (3.45)	4.9×10^6^(3.1×10^7^)
*Lamium purpureum*	2	Cotyledon stage	- 0.49 (1.78)	8.46 (137)	4.0×10^4^ (4.9×10^5^)
		Two-leaf stages	0.41 (0.16)	0.91 (0.09)	0.91 (0.09)
		Four-leaf stages	0.28 (0.18)	31.4 (4.13)	273 (444)
*Myosotis arvensis*	1	Cotyledon stage	0.91 (0.39)	1.39 (0.15)	0.21 (0.15)
		Two-leaf stages	0.94 (0.35)	7.43 (1.11)	0.87 (0.46)
		Four-leaf stages	0.71 (0.26)	12.0 (1.56)	1.42 (0.78)
*Myosotis arvensis*	2	Cotyledon stage	1.37 (0.70)	1.88 (0.20)	0.25 (0.13)
		Two-leaf stages	1.11 (0.37)	4.82 (0.51)	0.39 (0.15)
		Four-leaf stages	0.86 (0.20)	10.77 (0.96)	10.8 (0.96)
*Plantago major*	1	Cotyledon stage	-0.49 (1.78)	8.46 (137)	4.0×10^4^ (4.9×10^5^
		Two-leaf stages	1.36 (0.80)	2.74 (0.50)	0.89 (0.41)
		Four-leaf stages	0.78 (0.33)	15.4 (1.90)	0.40 (0.24)
*Plantago major*	2	Cotyledon stage	-0.49 (1.78)	8.46 (137)	4.0×10^4^ (4.9×10^5^
		Two-leaf stages	1.40 (2.96)	4.30 (0.57)	0.10 (0.33)
		Four-leaf stages	0.78 (0.33)	15.4 (1.90)	040 (0.24)
*Rumex crispus*	1	Cotyledon stage	1.94 (1.13)	7.33 (1.11)	4.18 (1.89)
		Two-leaf stages	1.19 (1.16)	21.89 (5.00)	3.25 (1.99)
		Four-leaf stages	0.37 (0.64)	37.1 (6.65)	838 (5008)
*Rumex crispus*	2	Cotyledon stage	1.99 (0.89)	4.70 (0.48)	0.39 (0.09)
		Two-leaf stages	3.27 (1.11)	30.4 (1.58)	4.08 (0.47)
		Four-leaf stages	1.15 (0.83)	32.1 (3.32)	12.5 (4.64)
*Sonchus oleraceus*	1	Cotyledon stage	0.99 (0.30)	8.61 (0.759	0.29 (0.13)
		Two-leaf stages	1.20 (0.38)	29.6 (3.34)	0.99 (0.31)
		Four-leaf stages	0.73 (0.27)	56.3 (4.69)	8.47 (2.71)
*Sonchus oleraceus*	2	Cotyledon stage	3.23 (2.26)	10.9 (0.97)	0.34 (0.06)
		Two-leaf stages	1.48 (0.35)	19.5 (1.76)	1.23 (0.28)
		Four-leaf stages	3.48 (2.67)	41.9 (3.32)	11.8 (2.3)
*Stellaria media*	1	Cotyledon stage	7.27 (NaN)	4.20 (0.60)	0.35 (NaN)
		Two-leaf stages	0.84 (0.35)	15.4 (2.49)	2.21 (1.37)
		Four-leaf stages	0.56 (0.25)	10.8 (1.21)	8.05 (5.19)
*Stellaria media*	2	Cotyledon stage	7.27 (NaN)	4.20 (0.60)	0.35 (NaN)
		Two-leaf stages	0.95 (0.26)	11.2 (1.16)	0.88 (0.31)
		Four-leaf stages	0.85 (0.55)	12.4 (2.15)	6.09 (4.56)
*Veronica persica*	1	Cotyledon stage	1.46 (0.46)	3.26 (0.38)	0.68 (0.20)
		Two-leaf stages	0.24 (0.44)	9.93 (2.46)	591 (4776)
		Four-leaf stages	0 (0)	0.007 (0.188)	NaN (NaN)
*Veronica persica*	2	Cotyledon stage	1.46 (0.46)	3.26 (0.38)	0.68 (0.20)
		Two-leaf stages	0.23 (0.13)	7.75 (0.63)	0.46 (1.10)
		Four-leaf stages	0.41 (0.28)	14.6 (2.29)	109 (146)
*Viola arvensis*	1	Cotyledon stage	1.05 (2.10)	1.98 (0.18)	0.03 (0.17)
		Two-leaf stages	0.99 (0.34)	6.05 (0.65)	0.38 (0.17)
		Four-leaf stages	2.26 (0.81)	6.13 (0.67)	6.85 (1.35)
*Viola arvensis*	2	Cotyledon stage	1.05 (2.10)	1.98 (0.18)	0.03 (0.17)
		Two-leaf stages	1.56 (0.41)	1.88 (0.07)	0.22 (0.05)
		Four-leaf stages	0.93 (0.34)	3.58 (0.48)	1.64 (0.80)

*b*: shape parameter; *d*: upper limit; *e*: inflection point of the curve. *NaN, not available.

Standard Errors (SE) in parentheses.

**Table 3:** Estimated ED_10,_ ED_50,_ and ED_90_ values corresponding to the dose reducing the growth by 10, 50, and 90 %, respectively.

Plant species	Exp.	Growth stage	ED_10_ (SE)	ED_50_ (SE)	ED_90_ (SE)
*Alopecurus myosuroides*	1	One-leaf stage	452 (8757)	4×10^4^ (5*10^5^)	3×10^6^ (8×10^7^)
		Two-leaf stage	0.09 (0.07)	0.26 (0.07)	0.71 (0.17)
		Three-leaf stage	0.26 (0.12)	0.69 (0.14)	1.83 (0.64)
*Alopecurus myosuroides*	2	One-leaf stage	0.002 (0.010)	0.032 (0.08)	0.50 (0.28)
		Two-leaf stage	0.09 (0.07)	0.26 (0.07)	0.71 (0.17)
		Three-leaf stage	0.64 (0.72)	0.76 (0.20)	0.90 (0.55)
*Beta vulgaris*	1	Cotyledon stage	0.13 (0.08)	0.40 (0.09)	1.19 (0.46)
		Two-leaf stage	0.05 (0.07)	1.24 (0.58)	28.9 (24.2)
		Four-leaf stage	0.05 (0.17)	14.9 (17.5)	14.9 (17.5)
*Beta vulgaris*	2	Cotyledon stage	0.29 (0.43)	0.38 (0.07)	0.50 (0.54)
		Two-leaf stages	0.47 (1.09)	2.75 (1.90)	16.0 (18.2)
		Four-leaf stages	12.2 (2.04)	15.1 (9.91)	18.6 (27.08)
*Erodium dissectum*	1	Cotyledon stage	0.11 (0.11)	0.35 (0.10)	1.08 (0.62)
		Two-leaf stages	0.08 (0.07)	0.61 (0.22)	4.62 (2.48)
		Four-leaf stages	1.03 (0.69)	3.94 (1.36)	15.1 (9.96)
*Erodium dissectum*	2	Cotyledon stage	1.26 (0.68)	1.52 (0.17)	1.84 (0.62)
		Two-leaf stages	0.13 (0.07)	0.61 (0.12)	2.85 (1.00)
		Four-leaf stages	1.65 (0.82)	3.57 (0.70)	7.73 (2.97)
*Geranium molle*	1	Cotyledon stage	0.002 (0.007)	0.05 (0.09)	1.24 (0.97)
		Two-leaf stages	0.13 (0.07)	0.61 (0.12)	2.85 (0.10)
		Four-leaf stages	0.05 (0.04)	1.04 (0.35)	21.2 (11.3)
*Geranium molle*	2	Cotyledon stage	0.01 (0.02)	0.15 (0.11)	1.77 (0.96)
		Two-leaf stages	0.05 (0.04)	0.28 (0.09)	1.55 (0.62)
		Four-leaf stages	2.09 (1.49)	5.49 (1.29)	14.4 (5.82)
*Lamium purpureum*	1	Cotyledon stage	452 (8757)	4.0×10^4^ (4.9×10^5^)	3.6×10^6^ (7.8×10^7^)
		Two-leaf stages	0.001 (0.005)	1.09 (0.80)	707 (1709)
		Four-leaf stages	69.2 (810)	4.9×10^7^ (3.1×10^8^)	3.48*10^13^ (2.4*10^14^)
*Lamium purpureum*	2	Cotyledon stage	0.15 (0.10)	0.68 (0.20)	3.07 (1.37)
		Two-leaf stages	0.004 (0.009)	0.82 (0.55)	176 (323)
		Four-leaf stages	0.11 (0.50)	273 (444)	6.7×10^5^ (4.1×10^6^)
*Myosotis arvensis*	1	Cotyledon stage	0.02 (0.03)	0.21 (0.15)	2.28 (1.40)
		Two-leaf stages	0.08 (0.10)	0.87 (0.46)	8.97 (6.62)
		Four-leaf stages	0.06 (0.10)	1.42 (0.78)	31.5 (32.4)
*Myosotis arvensis*	2	Cotyledon stage	0.05 (0.06)	0.25 (0.13)	1.23 (0.62)
		Two-leaf stages	0.05 (0.05)	0.39 (0.15)	2.85 (1.46)
		Four-leaf stages	0.14 (0.12)	1.75 (0.57)	22.5 (12.2)
*Plantago major*	1	Cotyledon stage	452 (8757)	4.0×10^4^ (5.0×10^5^)	3.5×10^6^ (7.8×10^7^)
		Two-leaf stages	0.18 (0.19)	0.89 (0.41)	4.47 (4.71)
		Four-leaf stages	0.02 (0.04)	0.40 (0.24)	6.62 (5.93)
*Plantago major*	2	Cotyledon stage	452 (8757)	4.0×10^4^ (5.0×10^5^)	3.5×10^6^ (7.8×10^7^)
		Two-leaf stages	0.02 (0.13)	0.10 (0.33)	0.47 (0.37)
		Four-leaf stages	1.35 (1.01)	4.18 (1.89)	13.0 (11.1)
*Rumex crispus*	1	Cotyledon stage	0.12 (0.08)	0.36 (0.09)	1.08 (0.43)
		Two-leaf stages	0.51 (1.19)	3.25 (2.00)	20.7 (28.3)
		Four-leaf stages	2.26 (13.0)	838 (5008)	3.1×10^5^ (4.9×10^6^)
*Rumex crispus*	2	Cotyledon stage	0.12 (0.08)	0.36 (0.09)	1.08 (0.43)
		Two-leaf stages	2.09 (0.54)	4.08 (0.47)	7.98 (1.98)
		Four-leaf stages	1.85 (2.43)	12.5 (4.64)	85.24 (131)
*Sonchus oleraceus*	1	Cotyledon stage	0.03 (0.03)	0.29 (0.13)	2.74 (1.28)
		Two-leaf stages	0.16 (0.12)	0.99 (0.31)	6.17 (3.59)
		Four-leaf stages	0.42 (0.50)	8.47 (2.71)	171 (196)
*Sonchus oleraceus*	2	Cotyledon stage	0.17 (0.11)	0.34 (0.06)	0.67 (0.24)
		Two-leaf stages	0.28 (0.13)	1.23 (0.28)	5.44 (2.02)
		Four-leaf stages	6.26 (3.34)	11.8 (2.35)	22.1 (11.4)
*Stellaria media*	1	Cotyledon stage	0.25 (NaN)	0.35(NaN)	0.47 (NaN)
		Two-leaf stages	0.16 (0.21)	2.21 (1.38)	30.1 (37.1)
		Four-leaf stages	0.16 (0.28)	8.05 (5.20)	412 (789)
*Stellaria media*	2	Cotyledon stage	0.26 (NaN)	0.35 (NaN)	0.47 (NaN)
		Two-leaf stages	0.09 (0.08)	0.88 (0.32)	8.83 (4.83)
		Four-leaf stages	0.46 (0.82)	6.09 (4.56)	80.9 (151)
*Veronica persica*	1	Cotyledon stage	0.15 (0.10)	0.68 (0.20)	3.07 (1.36)
		Two-leaf stages	0.07 (0.73)	591 (4776)	5.2 ×10^6^ (1.2×10^9^)
		Four-leaf stages	2×10^-4^ (NaN)	23.82 (032)	0.007 (0.19)
*Veronica persica*	2	Cotyledon stage	0.15 (0.10)	0.68 (0.20)	3.07 (1.36)
		Two-leaf stages	2×10^-5^ (2.2×10^-5^)	0.46 (1.10)	8052 (30117)
		Four-leaf stages	0.54 (1.82)	109 (146)	2.2×10^5^ (9.5×10^4^)
*Viola arvensis*	1	Cotyledon stage	0.004 (0.036)	0.03 (0.17)	0.24 (0.49)
		Two-leaf stages	0.04 (0.05)	0.38 (0.17)	3.52 (2.04)
		Four-leaf stages	2.59 (1.15)	6.85 (1.35)	18.1 (6.34)
*Viola arvensis*	2	Cotyledon stage	0.003 (0.036)	0.03 (0.17)	0.24 (0.49)
		Two-leaf stages	0.05 (0.03)	0.22 (0.05)	0.90 (0.18)
		Four-leaf stages	0.15 (0.19)	1.64 (0.80)	17.4 (11.4)

*NaN, not available; SE, Standard Error. Standard Errors (SE) in parentheses.

## 4 Discussion

For all plant species, the youngest growth stage was the most sensitive to irradiation and the largest the least. The plant species showed a large difference in response to the dose range, and plants from the same species also reacted differently to the same dose probably for the following reasons: 1) the plants may not have been hit exactly the same place; 2) there were slight morphological differences; 3) minor variations in the development; and 4) leaves partly covered the apical meristem of some plants. Furthermore, the individual experiments were conducted over an extended period with different light conditions and some temperature variation, and, therefore, the 21 days of growth of some species resulted in significant differences in biomass production between experiments 1 and 2; for example, if one was done in summer and one in the late fall, the biomass productions were different after 21 days because the plants have been exposed to varying degree days. The variation is reflected in the standard errors of the estimates of the model parameters (**Table 2**) and the estimated standard errors of the ED_10_, ED_50_ and ED_90_ values (**Table 3**).

### 4.1 The grass weed

A pre-experiment showed that it was impossible to get a good effect of the laser treatment on *A. myosuroides* from an angle of 90°. The apical meristem of grasses is placed close to or below the ground, and the leaves protect the meristem. Burning a hole in a leaf does not prevent a plant from recovering and regrowing. Therefore, the angle was changed to 45°, and the beam was directed towards the base of the plants cutting the leaves and resulting in reduced or no regrowth (**Figure 2**). A similar technique was used by Rakhmatulin and Andreasen (2020) on the perennial grass *Elymus repens* Desv. Es Nevski. and by Heisel et al. (2001) on *Lolium perenne* L., who used an angle of 15°.

At the first leaf stage, *A. myosuroides* was seriously affected by all laser doses, and no biomass was produced afterwards (**Figure 2**). At the two-leaf stage, only the two smallest doses resulted in minor regrowth. At the three-leaf stage, a dose of 3.2 J mm^-2^ prevented regrowth. New methods that can replace herbicides to control this winter annual grass weed are in demand (Klauk and Petersen, 2023) because herbicide- and multiple herbicide-resistant biotypes (Heap, 2024) are widespread, and because the large areas with autumn sown annual crops in Europe create good condition for the species. As demonstred by our results low laser doses efficiently control it. In cereals, it is essential to laser weed early in the season before the cereals cover the grass weeds. Later, it might be challenging to find the plants, and the risk of hitting crop plants will increase. However, if the laser accidentally hits crop leaves, it will not do much harm as the cereal will just regrow.

### 4.2 The dicot crop

At the cotyledon stage, *B. vulgaris* was also sensitive to low laser doses (**Figure 3**). As the crop continued to grow, for example at 2- and 4-leaf stages, only the highest doses resulted in major reduced regrowth. The relatively thick sugar beet leaves make them less sensitive to laser irradiation, which can be an advantage in a sugar beet field, if the crop accidentally is hit by the laser beam.

### 4.3 The dicot weeds

For all the dicotyledons weed species, the highest dose (12.7 J mm^-2^) irradiated at the cotyledon stage also prevented regrowth, and most of the species were seriously harmed by the laser even at low dosages, resulting in low biomass production after three weeks (**Figures 4**−**11**).

When dicots were treated with the highest dose at the two-leaf stage, most of the species were unable to produce biomass, but the species reacted differently to the small doses. Some species with small and thin leaves, like *G. molle* (**Figure 5**), *P. major* (**Figure 8**), and *V. arvensis* (**Figure 13**) were the most sensitive, while *R. crispus* (**Figure 9**), a species with relatively large leaves, was less sensitive at the 2-leaf stage. The highest laser dose only prevented *Stellaria media* from regrowing in one of the experiments (**Figure 11**).

**Figure 14:**
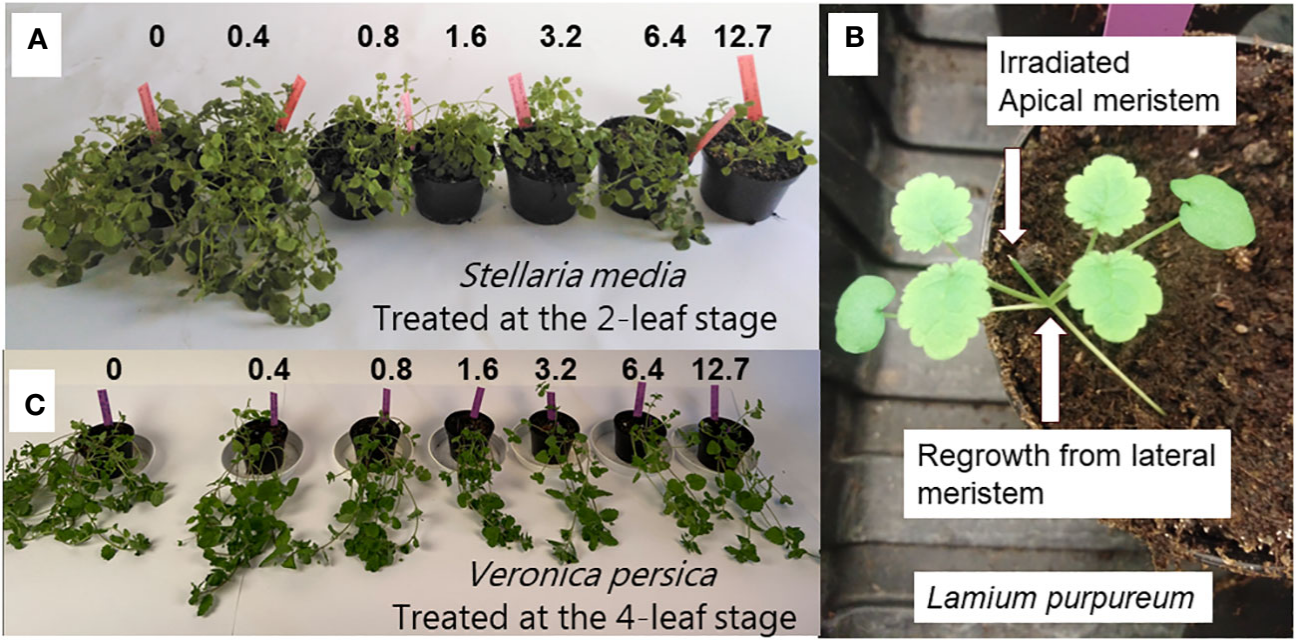
**(A)** Regrowth of *Stellaria media* plants three weeks after laser treatments on the 2-leaf stage. **(B)** Regrowth of *Lamium purpureum* plants from lateral meristems at the base of the cotyledons. **(C)** Regrowth of *Veronica persica* plants three weeks after laser treatment on the four-leaf stage. The unit of the doses is J mm^-2^.

Increasing laser doses at the 4-leaf stage affected most of the dicot weed species, but the effect was much less pronounced than at the 2-leaf stage. *Lamium purpureum* (**Figure 14**)*, S. media*, and *V. persica* quickly sprouted from lateral meristems at the base of the cotyledons when the laser killed the apical meristem. The epicotyl grew fast and moved the apical meristem a certain distance from the cotyledons as the plants developed the first two permanent leaves. Therefore, the lateral meristems avoided being hit by the laser beam (**Figure 14**). Even the highest dose (12.7 J mm^-2^) did not reduce the biomass production much compared with the untreated plants (**Figure 14**). Therefore, plants should preferably be irradiated before the epicotyl develops. Other weed species can regrow from lateral meristems on the hypocotyl. Examples are species in the Euphorpia genus like *E. exigua, E. peplus*, and *E. helioscopia* (Haas and Laursen, 1975). Suppose the laser beam kills the apical meristem between the cotyledons; in that case the plant may survive by sprouting from the lateral meristems on the hypocotyls as long as sufficient resources are stored in the tissue. However, the treatment will always delay the growth and reduce the weeds’ ability to compete against fast-growing crop plants.

### 4.4 Time window for laser weeding

The experiments showed the best effect when plants were treated at the earliest growth stages. The same counts for other weed control methods like herbicide application (Streibig, 1988), mechanical weeding (Håkansson, 2003), and flame weeding (Ascard, 1994), but the period when laser weeding is efficient is shorter than for some herbicides that can kill plants with more than four permanent leaves. If the weather or soil conditions make driving impossible in the fields while the weed plants are small, deployment of laser weeding would be challenging because controlling large plants would require a larger beam diameter to cover the meristem and significantly higher energy doses than those used in our experiments. Furthermore, it might be necessary to kill several meristems on the same plant.

### 4.5 Laser effect

If the largest dose (12.7 J mm^-2^) is necessary to kill weeds, it would take 0.8 s plant^-1^ with the 50 W fibre laser (**Equation 1**). With 300 weeds m^-2^, it would take 4 minutes to control 1 m^2^, resulting in a very slow driving speed. Therefore, it is necessary to use more powerful lasers in autonomous laser vehicles. In the WeLASER project (https://welaser-project.eu/), the intention was to install 500 W fibre lasers, resulting in a significantly higher weeding capacity (Emmi et al., 2024). In row crops, several passes are usually necessary to control weeds because new plants are emerging continuously until the crop covers the ground. In fields with high weed densities, it may be necessary to use chemical or mechanical weed control for the first pass, and then laser weed the second, third, and fourth passes. Using herbicides or mechanical weeding between the rows and laser weeding in the row would also increase the weeding capacity and, hence, the driving speeds. In contrast to mechanical weeding, the laser can control weeds very close to the crop plants without damaging the roots and leaves of the crop plant. When considering large areas, a fleet of robots may be necessary, to control weeds timely (Gonzalez-de-Santos et al., 2017).

### 4.6 Laser beam diameter

The beam diameter used in our studies was 2 mm. A larger bean diameter may be more appropriate to ensure a high hit rate under field conditions. The hit rate is affected by the laser vehicle’s speed and the soil surface’s bumpiness, which may causes vibrations. However, a larger beam diameter will require more energy to achieve the same heating effect on plants, as illustrated in laser weeding experiments with *Elymus repens* (Rakhmatulin and Andreasen, 2020).

### 4.7 Laser types

A thulium-doped fibre laser with a wavelength of 2 µm has the advantage of penetrating deeper into the plant tissue than a CO_2_ laser and heating the water in the cells (Wieliczka et al., 1989). Therefore, it is considered more efficient than a CO_2_ laser. Doing the same experiments with a CO_2_ laser or other laser types (Andreasen et al., 2022) would probably result in other dose-response relationships. To our knowledge, plant experiments have never been done comparing the effects of the two laser types.

### 4.8 Laser safety

We used a collimated laser beam to precisely expose the plants to the desired dose independent of the distance to the target, which could vary due to variations in plant heights. If reflecting materials are hit with a collimated laser beam, such as a piece of glass, metal, or a stone, the beam may escape the target area and burn or blind humans or animals (dogs, hares, rabbits, etc.). Therefore, collimated laser beams are not suitable for laser weeding robots. The laser beams should only be focused and concentrated on the weed seedlings’ meristem. If it then escapes the target area, the beam will be spread in a cone, and the risk of harming humans, animals, and crop plants would be significantly reduced due to the lower dose per area. Therefore, only organisms (e.g., aphids) placed precisely in the focus point would receive the dose determined for the target plant. The further away from the focus point, the lower the dose an organism would receive, and the less harmful would the exposure be.

## 5 Conclusion

All species were most susceptible to laser irradiation at the youngest growth stage, and it decreased with increasing plant size. The plant species showed large differences in the response to the dosages. If artificial intelligence can be used to recognise the different weed species and their size in the field, the laser dose can be adjusted after each plants developmental stage and susceptibility to optimise the energy consumption. Within the dose range 0.4−12.7 J mm^-2^, *A. myosuroides* was well-controlled, and laser weeding could be a way to overcome the increasing problems with herbicide-resistant biotypes. Sugar beet was less susceptible than most of the weeds, which can be considered as an advantage if it is accidentally hit by the laser beam. Growth habits influenced the species’ ability to regrow after laser exposure. *Lamium purpureum*, *S. media*, and *V. persica* rapidly established an epicotyl, moving the two first permanent leaves away from the apical meristem. Therefore, the lateral meristems at the base of the cotyledons avoided to be killed and rapidly started to grow. Laser treatment should be conducted at the cotyledon or two-leaf stage to optimize energy consumption and achieve the best effect. In the future, the laser effect in the field should be tested. Also, experiments with a range of laser beam diameters need to be done, as the beam diameter is detrimental for the hit rate of a moving autonomous laser vehicle.

## Data availability statement

The raw data supporting the conclusions of this article will be made available by the authors, without undue reservation.

## Author contributions

CA: Conceptualization, Data curation, Formal analysis, Funding acquisition, Investigation, Methodology, Project administration, Resources, Software, Supervision, Validation, Visualization, Writing – original draft, Writing – review & editing. EV: Data curation, Formal analysis, Investigation, Methodology, Validation, Visualization, Writing – original draft, Writing – review & editing. NS: Data curation, Formal analysis, Investigation, Methodology, Validation, Visualization, Writing – original draft, Writing – review & editing.
